# Methylglyoxal-derived hydroimidazolone residue of plasma protein can behave as a predictor of prediabetes in Spontaneously Diabetic Torii rats

**DOI:** 10.14814/phy2.12477

**Published:** 2015-08-11

**Authors:** Si Jing Chen, Chiwa Aikawa, Risa Yoshida, Toshiro Matsui

**Affiliations:** Division of Bioscience and Bioenvironmental Sciences, Faculty of Agriculture, Graduate School of Kyushu UniversityFukuoka, Japan

**Keywords:** Advanced glycation end products, Spontaneously Diabetic Torii rats, impaired glucose tolerance

## Abstract

Prediabetes, typically defined as impaired glucose tolerance and/or impaired fasting blood glucose, is a high-risk state of developing diabetes. The association of diabetes-related metabolites with prediabetes has not been investigated intensively. This study aimed to get insights into the metabolic behaviors of some typical diabetes-related metabolites in plasma of male Spontaneously Diabetic Torii (SDT) rats during pathogenic progress of diabetes and to assess in vivo if the variation in these metabolites related to the progression of prediabetic stage. To address this question, SDT rats and Sprague Dawley (SD) rats as control were maintained from the age of 7 to 25 weeks. Five typical advanced glycation end products (AGEs) residue of plasma protein and their free adducts were determined by liquid chromatography with tandem mass detection over the duration of the investigation. The SDT rats exhibited impaired glucose tolerance since the age of 12 weeks and developed diabetes with significantly elevated fasting glucose levels after 22 weeks. At the prediabetic stage (12–21 weeks), no significant differences were observed on AGE-free adducts levels of SDT rats compared with SD rats. However, methylglyoxal-derived hydroimidazolone (MG-H1) residue contents of plasma protein were significantly elevated in SDT rats at the age of 16 weeks, whereas other AGE residues of plasma protein did not show marked difference. The present study has revealed significant increase in MG-H1 residue content of plasma protein at the prediabetic stage of a spontaneously diabetic rat model, irrespective of unaltered fasting blood glucose and constant plasma levels of other AGEs.

## Introduction

Metabolic diseases are usually present for years before becoming clinically apparent. Prediabetes, typically defined as impaired glucose tolerance and/or impaired fasting blood glucose, is a high-risk state of developing diabetes (Tabák et al. 2012). The onset of type 2 diabetes can be prevented or delayed by early interventions such as lifestyle changes and therapeutic interventions at the prediabetic stage (Nathan et al. 2007, 2009). Current clinical indicators, such as fasting and postprandial blood glucose and hemoglobin A_1C_ (HbA1c), can be helpful in increased risks of diabetes, prediabetes, impaired fasting glucose, and impaired glucose tolerance (American Diabetes Association, 2015). However, given the availability of effective interventions for preventing the increased burden of diabetes worldwide, earlier identification of individuals at risk is particular crucial (Knowler et al. 2002; The Diabetes Prevention Program Research Group, 2005).

Animal models of diabetes are important resources for the pathophysiological and therapeutic researches on diabetes and diabetic complications (Rees and Alcolado 2005). Particularly, animals which genetically develop diabetes in a spontaneous pattern have contributed to the research on human diabetes and diabetic complications because genetic factors are known to play a crucial role in the development of type 2 diabetes (Ktorza et al. 1997) The male Spontaneously Diabetic Torii (SDT) rat, an inbred strain of Sprague Dawley (SD) rat, was established as a model of nonobese spontaneous diabetes, and develops hyperglycemia after 20 weeks of age with an incident rate reaching 100% at 40 weeks of age (Masuyama et al. 2004). In addition, recent studies demonstrated that diabetes in the SDT rat has a polygenic basis and they develop severe diabetic complications which resemble human type 2 diabetes (Shinohara et al. 2000; Sasase et al. 2006). Taking advantage of these features, SDT rats should be a suitable model for studies on pathogenic progress of diabetes.

Glycation metabolites such as advanced glycation end products (AGEs), which are generated from the modification of proteins with high reactive carbonyl intermediates from glucose, have been revealed to exert considerable effects in diabetes and its complications (Goh and Cooper 2008). Moreover, clinical study showed that the increase in glycation metabolites is related to the progression of diabetes (Ahmed and Thornalley 2007). Herein, we hypothesize that altered plasma AGEs levels may be served as diagnostic biomarkers of prediabetes and enable preventive interventions. In previous report, we proposed a liquid chromatography/tandem mass spectrometry (LC-MS/MS) method in combination with 2,4,6-trinitrobenzene sulfonate (TNBS) derivatization technique for high-sensitive detection of AGE adducts at >0.16 nmol/L (Hashimoto et al. 2013) and have applied it to the determination of plasma levels of AGE-free adducts in young Wistar rats under an acute hyperglycemia condition (Chen et al. 2015). The proposed TNBS-MS method allowed us to (i) reliably determine AGE levels in biological samples with the aid of stable isotopic internal standards (IS) and (ii) investigate their metabolic behavior in living animals, because of low plasma sample volume (∼50 *μ*L) required from the tail vein.

**Figure 1:**
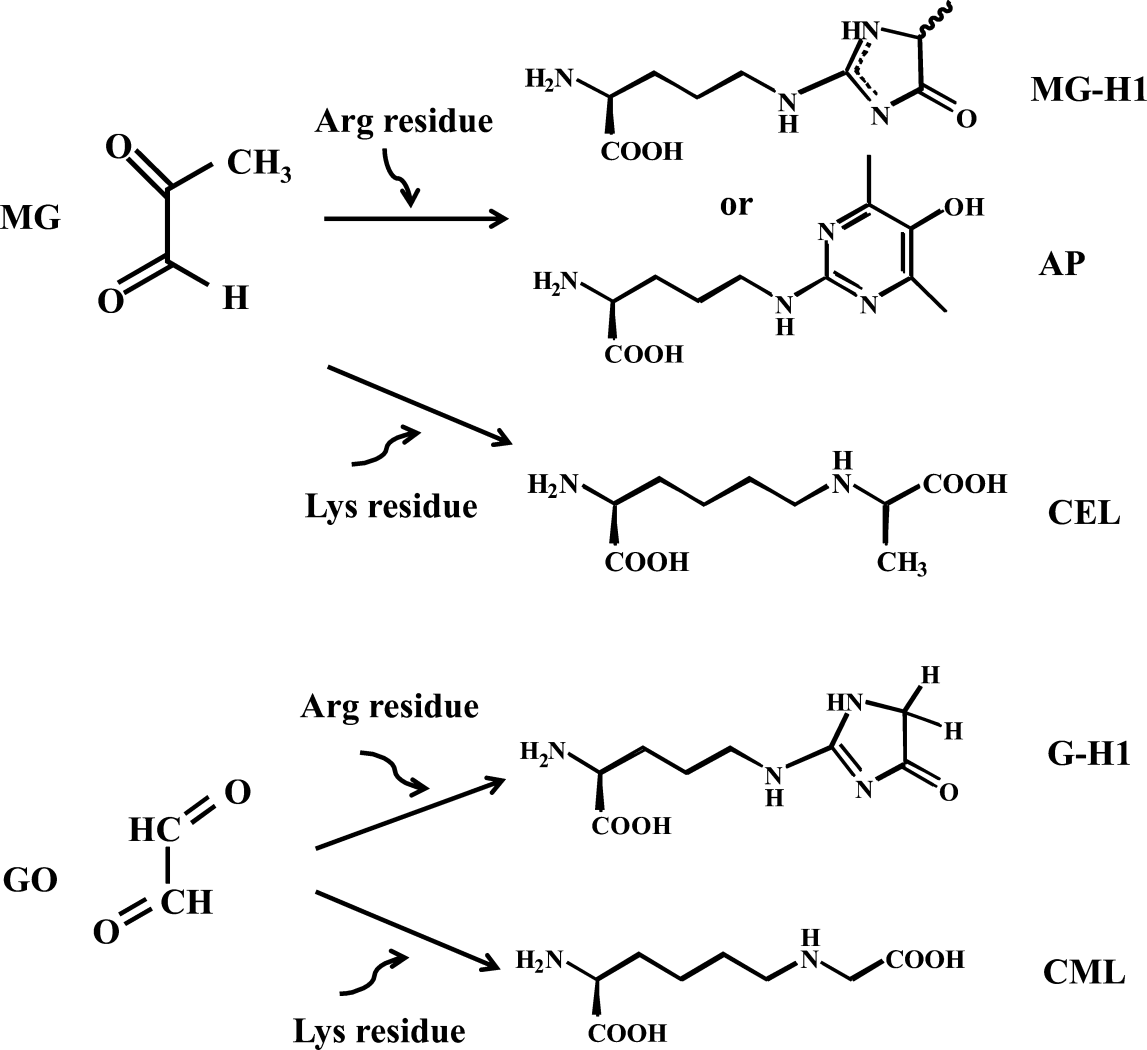
Formation of five typical advanced glycation end products (AGEs) by the reaction of MG or GO with arginine or lysine residue.

The aim of the present study was to get insights into the metabolic behavior of some typical diabetes-related metabolites in the plasma samples of SDT rats during pathogenic progress of diabetes and to assess in vivo if the variation in these metabolites related to the progression of prediabetic stage. Five widely investigated AGE adducts, for example, methylglyoxal-derived hydroimidazolone (MG-H1), glyoxal-derived hydroimidasolone (G-H1), *N*^*ε*^-(1-carboxyethyl)-lysine (CEL), *N*^*ε*^-carboxy methyl-lysine (CML), and argpyrimidine (AP) were targeted in present study (Fig.1).

## Methods

### Ethical approval

All animal experiments were in accordance with the Guidance for Animal Experiments in Faculty of Agriculture and in the Graduate Course of Kyushu University and the Law (No. 105, 1973) and Notification (No. 6, 1980 of the Prime Minister’s Office) of the Japanese Government. All experiments were reviewed and approved by the Animal Care and Use Committee of Kyushu University (Permit Number: A24-051).

### Animals and experimental protocol

Five male SDT rats (7-week-old) and age-matched male SD rats as control animals were purchased from CLEA Japan (Tokyo, Japan). Rats were acclimatized under laboratory conditions (21 ± 1°C, 55.5 ± 5% humidity, a 12 h light–dark cycle) for 1 week before experiments and given free access to a moderate fat diet and distilled water over 18 weeks. Blood samples were collected from the tail vein every week after a 16-h fasting while the blood glucose levels were checked. Plasma was separated by centrifugation (4°C, 3500*g*, 15 min) and stocked at −30°C for further analysis.

### Oral glucose tolerance test (OGTT)

Oral glucose tolerance test was performed every 4 weeks to evaluate glucose tolerance of rats. The rats were fasted overnight (16 h) prior to a single oral administration of glucose solution (2 g/kg body weight). Blood glucose testing was performed before the gavage and at certain time points after administration (0, 30, 60, 90, 120 min). Body weight and food consumption were characterized.

### Measurements of blood glucose and plasma insulin

Blood glucose levels (BGL) were determined by blood glucose test meter with disposable blood glucose sensor (Glutest Pro, Sanwa Chemical Research Co., Tokyo, Japan). Plasma insulin levels were measured using a rat insulin enzyme-linked immunosorbent assay (ELISA) kit (Shibayagi Co., Ltd., Gunma, Japan) according to the manufacturer’s instructions. The homeostasis model assessment of insulin resistance (HOMA-IR) was calculated using the following formula: HOMA-IR = fasting glucose × fasting insulin/22.5 (Matthews et al. 1985).

### Determination of AGEs by LC-MS/MS

Advanced glycation end products were determined by LC-MS/MS in combination with TNBS derivatization technique according to our previous studies (Hashimoto et al. 2013; Chen et al. 2015). Analytes determined were: MG-H1, G-H1, CEL, CML, and AP.

Briefly, to 50 *μ*L plasma, 10 *μ*L of internal standard (IS) mixture (50 nmol/L MG-H1-*d3* and 500 nmol/L CEL-*d4*, PolyPeptide Laboratories France SAS, Strasbourg, France), and 150 *μ*L of 20% acetonitrile containing 0.1% formic acid (FA) was added, followed by centrifugation at 14 000*g* for 30 min at 4°C using a Millipore Amicon Ultra-0.5 centrifugal filter with a molecular weight cut of <3000 Da (Billerica, MA). The filtrate was evaporated to dryness, 50 *μ*L of 150 mmol/L TNBS solution (pH 8.5) was added to the sample, and incubated at 30°C for 30 min, then added 50 *μ*L of 0.2% FA to stop reaction. Plasma protein extracts were obtained and washed by three cycles of dilution and centrifugation in 20% acetonitrile containing 0.1% FA over the 3 kDa cut-off centrifugal filters. The protein concentrations of extracts were then measured by Lowry’s method using a DC protein assay kit (Bio-Rad Laboratories, Hercules, CA) and BSA was used as the standard. AGE residue contents of plasma protein were determined after exhaustive hydrolysis of protein extract (50 *μ*g, dissolved in 50 *μ*L of distilled water containing 500 nmol/L MG-H1-*d3* and 250 nmol/L CEL-*d4*) in 6 mol/L HCl at 110°C for 24 h in the presence of nitrogen according to the report by Mostafa et al. (2007) with slight modification. The hydrolysates were then derivatized by TNBS (pH 8.5, 30°C, 30 min) and subjected to LC-MS/MS analysis (Hashimoto et al. 2013). The estimates of AGE residues in plasma protein were presented by pmol/mg protein.

Samples were injected into an LC-MS/MS system (Agilent1200 HPLC; Agilent Technologies, Waldbronn, Germany) coupled with an Esquire6000 ESI-Ion Trap mass spectrometer (Bruker Daltonics, Bremen, Germany). Analytes were separated on a Waters 3 *μ*m Atlantis T3 column (2.1 × 100 mm) (Waters, Milford, MA) and detected by electrospray positive ionization multiple reaction monitoring LC-MS/MS. Monoisotopic isolations (*m/z*) of TNP-AGEs at the width of *m/z* 1.5 were 440.2 > 184.1, 426.0 > 152.1, 430.0 > 156.1, 416.0 > 142.0, 466.0 > 192.0, 443.1 > 187.1, and 434.0 > 160.0 for TNP-MG-H1, TNP-G-H1, TNP-CEL, TNP-CML, TNP-AP, TNP-MG-H1-*d3*, and TNP-CEL-*d4*, respectively.

### Determination of AGEs by ELISA

The Oxi Select AGE competitive ELISA kit (Cell Biolabs, Inc., San Diego, CA) was used to examine the total levels of AGEs in plasma, according to the manufacturer’s instructions.

### Statistical analysis

Statistical analysis was performed using the GraphPad Prism 5 software (GraphPad Software Inc., San Diego, CA). Data are expressed as mean ± SEM. One-way analysis of variance (ANOVA) was used to analyze the statistical differences in each group, followed by the Dunnett’s test for multiple comparisons. Other statistical evaluations were performed by the Student’s *t*-test. A *P* < 0.05 was considered significant.

## Results

### Characteristics of SD and SDT rats

Biological characteristics and parameters related to diabetes at different ages in SD and SDT rats were shown in Table1. The average body weight of male SDT rats was slightly higher than that of age-matched SD rats until 16 weeks of age and gradually decreased. A significant decrease in body weight was observed at 18 weeks of age (*P *<* *0.01). SDT rats were observed to have a significantly larger amount of food intake since 18 weeks of age (*P *<* *0.001). The fasting BGL in SDT rats significantly increased since 22 week of age (SD: 63.4 ± 1.2 vs. SDT: 87.5 ± 12.7 mg/dL, *P *<* *0.05). Besides, fasting plasma insulin concentrations in the SDT rats decreased significantly at the age of 24 weeks (*P *<* *0.001), with respect to SD control rats.

**Table 1:** Characteristics of the SD and SDT rats at different ages

Parameters	Rats	Age (weeks)				
		8	12	16	18	24
Body weight (g)	SD	218.7 ± 2.7	402.4 ± 6.6	500.6 ± 13.9	528.8 ± 13.7	595.2 ± 17.5
	SDT	298.7 ± 2.6***	436.3 ± 1.8**	499.0 ± 5.4	486.0 ± 4.9**	432.3 ± 4.8***
Food intake (g/day)	SD	23.4 ± 0.7	28.8 ± 0.4	28.8 ± 0.8	28.2 ± 0.4	26.6 ± 0.8
	SDT	27.4 ± 0.5	27.4 ± 0.5	29.3 ± 0.6	34.8 ± 0.8***	41.7 ± 2.1***
Fasting BGL (mg/dL)	SD	63.3 ± 1.8	62.8 ± 3.5	66.5 ± 3.4	68.1 ± 2.4	69.5 ± 2.6
	SDT	65.3 ± 1.6	70.5 ± 2.3	58.3 ± 4.1	69.6 ± 3.9	99.1 ± 15.2***
Insulin (*μ*U/mL)	SD	84.4 ± 0.3	36.1 ± 7.5	38.2 ± 5.1	38.5 ± 6.3	37.2 ± 5.9
	SDT	85.5 ± 1.0	62.4 ± 19.5	51.4 ± 5.9**	53.2 ± 22.1	18.9 ± 2.0***
OGTT-AUC_0–2 h_ (mg·h/dL)	SD	211.7 ± 8.1	194.2 ± 6.5	199.7 ± 4.4	–	198.6 ± 7.8
	SDT	330.8 ± 11.9***	445.6 ± 13.3***	527.7 ± 27.8***	–	765.3 ± 27.1***
HOMA-IR	SD	13.1 ± 0.5	5.4 ± 0.9	6.2 ± 0.8	6.6 ± 1.1	5.7 ± 0.8
	SDT	13.8 ± 0.4	10.5 ± 3.3	7.2 ± 0.5	8.6 ± 2.8	4.6 ± 0.8
Plasma AGEs^a^ (*μ*g/mL)	SD	126 ± 35	103 ± 25	140 ± 21	186 ± 38	102 ± 18
	SDT	134 ± 47	179 ± 79	113 ± 48	171 ± 25	98 ± 12

Data are mean ± SEM (SD, *n* = 5; SDT, *n* = 4)

a Plasma AGE levels were determined by a commercial AGE competitive ELISA kit. The dash (–) means no experiment performed.

Statistical differences were evaluated by Student’s *t*-test:

** *P* < 0.01

*** *P* < 0.001 versus the age-matched control SD rats.

### OGTT

**Figure 2:**
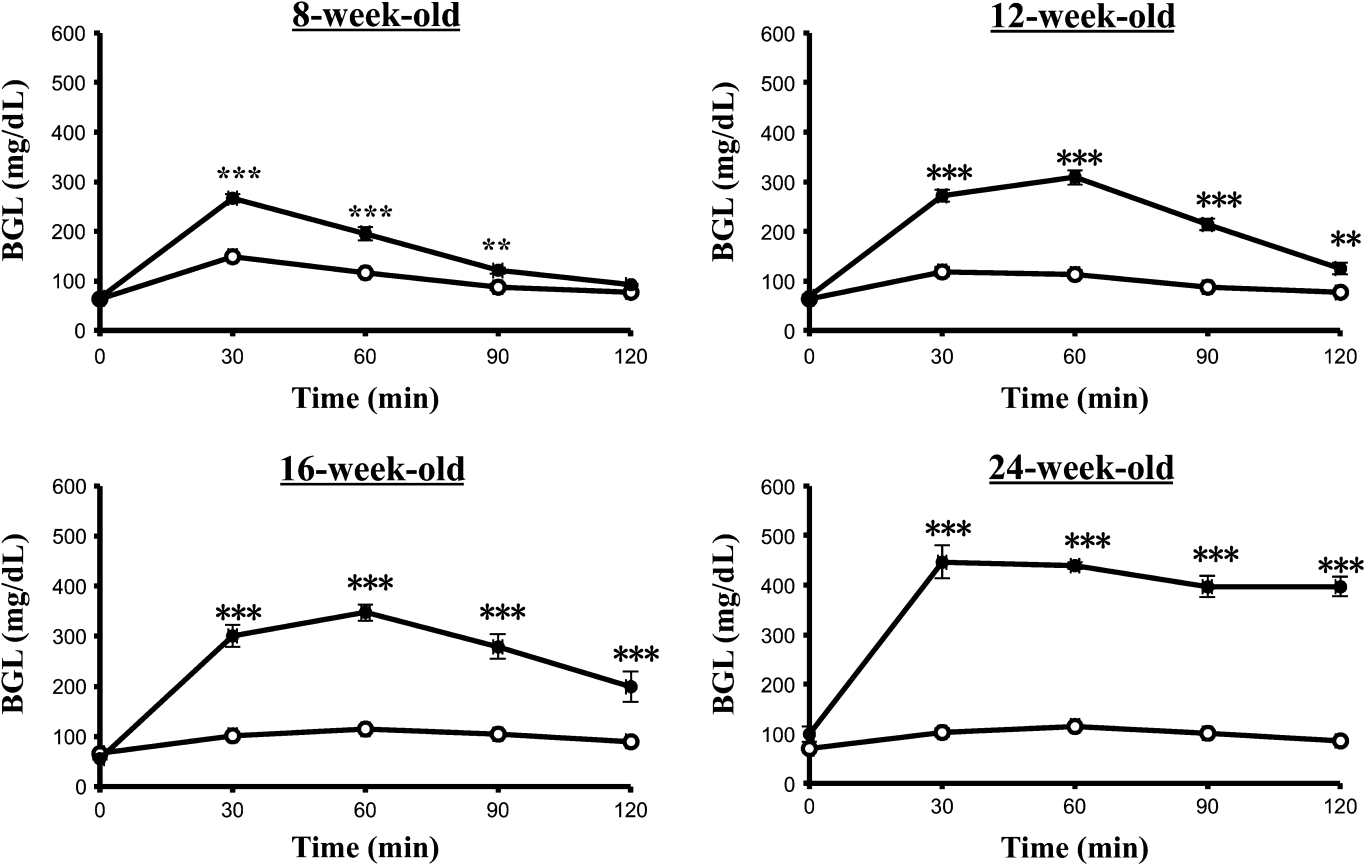
Blood glucose response during the OGTT (2 g/kg dose) in the Spontaneously Diabetic Torii (SDT) rats and the control SD rats. Experiments were performed after a 16-h fasting. Data are expressed as mean ± SEM (SD, *n* = 5; SDT, *n* = 4). Open and closed circles indicate the control SD rats and SDT rats, respectively. Significance: **P < 0.01 and ***P < 0.001 indicate statistical differences versus SD control rats at the same time point.

The results of the OGTT in SD and SDT rats at 8, 12, 16, and 24 weeks were shown in Figure2. Slight glucose intolerance was first noted in SDT rats at the age of 12 weeks. The blood glucose levels at 30, 60, 90, and 120 min after glucose administration in SDT rats (16-week-old) significantly (*P *<* *0.001) increased, compared with those in SD rats. At the age of 16, 20, and 24 weeks, the blood glucose levels were further elevated and sustained during the 2-h study period. The SDT rats had significantly greater area under the curve (AUC) than that of age-matched SD rats, while the AUC of glucose response in SDT rats increased with age (Table1). These results indicated that marked impaired glucose tolerance induced since 12 weeks of age in SDT rats.

The diabetic SDT rats were characterized at the age of 22 weeks by significantly elevated fasting glucose levels with respect to the control SD rats (*P *<* *0.05), accompanied with marked glucose intolerance and another typical diabetic symptoms (e.g., an increase in water and food consumption, body weight loss, and polyuria). The basal characteristics and diagnosis of diabetes of SDT rats were in agreement with a previous report (Masuyama et al. 2004). The prediabetic stage of SDT rats then was diagnostic from the age of 12 to 21 weeks in the present study (one of the five SDT rats did not develop diabetes within the duration of investigation).

### AGEs in the plasma of SDT and SD rats

**Figure 3:**
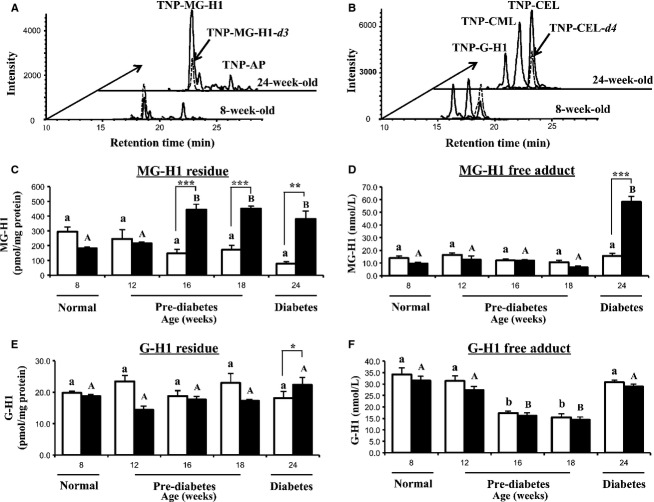
Arginine-derived hydroimidazolone residues and corresponding free adducts in plasma of SD and SDT rats at different ages. (A) Specimen chromatograms of MG-H1- and AP-free aducts in plasma of SDT rats at 8 and 24 weeks, (B) specimen chromatograms of G-H1-, CML-, and CEL-free adducts in plasma of SDT rats at 8 and 24 weeks, (C) MG-H1 residue, (D) MG-H1-free adduct, (E) G-H1 residue, and (F) G-H1-free adduct. Data are mean ± SEM (*n* = 4). Hollow bars, control SD rats; solid bars, SDT rats. Significance: statistical differences within group were evaluated using one-way analysis of variance (ANOVA), different letters represent the statistical differences at *P *< 0.05 among the means in each group by Dunnett’s test for multiple comparisons. *indicates significance of difference with respect to age-matched SD control rats by Student’s *t*-test, where one, two, and three symbols reflect *P < 0.05, **P < 0.01, and ***P < 0.001, respectively.

AGE residues were determined in HCl-hydrolysates of plasma protein and corresponding free adducts were detected in the filtrate of plasma. Specimen chromatograms of AGEs were given (Fig.3A and B), showing the successful detection of each target AGEs in rat plasma. The addition of ISs before HCl-hydrolysis of plasma proteins in this study could compensate the unexpected acid degradation of targets and ISs during HCl hydrolysis at 110°C for 24 h.

Arginine-derived hydroimidazolone residues are quantitatively important AGEs of plasma protein (Thornalley et al. 2003). MG-H1 residue content of plasma protein of SDT rats was increased significantly since the age of 16 weeks, compared with the age-matched SD rats (SD: 148.2 ± 25.9 vs. SDT: 444.2 ± 37.5 pmol/mg protein, *P *<* *0.001, Fig.3C). Besides, it also has given an over twofold increase, with respect to the baseline of SDT rats (8-week-old). The increasing tendency of MG-H1 residue in SDT rats was also confirmed in plasma samples treated by enzymes using pepsin, pronase E, aminopeptidase M, and prolidase (Ahmed et al. 2002) (data not shown), revealing that the effect of enzymatic or acid hydrolysis on MG-H1 residue content would be compensated in this study. As for MG-H1-free adduct concentration, the levels in SD and SDT rats kept stable levels until 18-week-old and, then, showed a marked increasing at the age of 24 weeks (SD: 15.7 ± 2.1 vs. SDT: 58.1 ± 4.4 nmol/L, *P *<* *0.001, Fig.3D). It has been reported that the exchange reaction of the MG moiety in MG-H1 (or -*d3*) with free Arg occurred and produced the authentic MG-H1 at mild physiological conditions (Thornalley 2006), but the rapid derivatization reaction of TNBS with amines (Cayot and Tainturier 1997) would compensate the authentic MG-H1 production or constant level of MG-H1-*d3* as IS in this study.

G-H1 residue contents of plasma protein of the SDT rats did not show significant difference with respect to those of SD rats from the age of 8 to 18 weeks, but have increased slightly at 24 weeks (SD: 18.1 ± 2.0 vs. SDT: 22.4 ± 2.4 pmol/mg protein, *P *<* *0.05, Fig.3E). There was a surprising similarity in G-H1-free adduct concentrations of SD and SDT (Fig.3F). The concentrations also showed remarkable resemblance of the variation tendency over the whole investigated duration.

**Figure 4:**
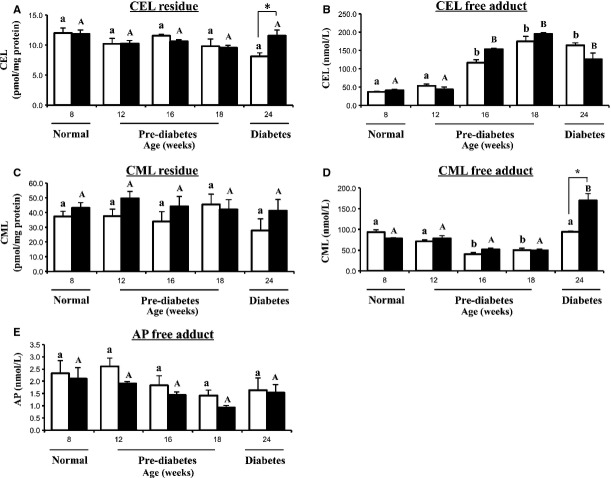
Advanced glycation end product (AGE) residues and AGE-free adducts in plasma of SD and Spontaneously Diabetic Torii (SDT) rats at different ages. (A) CEL residue, (B) CEL-free adduct, (C) CML residue, (D) CML-free adduct, and (E) AP-free adduct. Data are mean ± SEM (*n* = 4). Hollow bars, control SD rats; solid bars, SDT rats. Significance: symbol key as in Figure3.

CEL, a stable chemical modification of proteins, is formed by the Maillard reaction of lysine residues with MG (Ahmed et al. 1997). The CEL residue content of plasma protein of the SD and SDT rats were relatively stable over the investigated duration except for a slight but significant increasing of the CEL content in SDT rats at the age of 24 weeks of age (SD: 8.1 ± 0.9 vs. SDT: 11.6 ± 0.9 pmol/mg protein, *P *<* *0.05, Fig.4A). In contrast, the plasma concentrations of CEL-free adduct increased markedly both in SD and SDT rats, with respect to the baseline levels (8-week-old). At the diabetic stage (24-week-old), CEL concentration of SDT rats did not show significant differences, with respect to that of SD rats (Fig.4B).

CML has been suggested to present a marker of oxidative stress and oxidative damage to proteins in aging and diabetes (Schleicher et al. 1997). The CML residue content of plasma protein of the SD and SDT rats did not show the significant differences over the investigation duration (Fig.4C). However, the plasma concentration of CML-free adducts in SDT rats at the age of 24 week increased approximately twofold, with respect to the baseline of SDT rats (8-week-old). Besides, it also showed higher level compared with age-matched SD rats (SD: 94.2 ± 1.8 vs. SDT: 169.9 ± 17.0 nmol/L, *P *<* *0.05, Fig.4D).

AP, a major fluorescent product of the Maillard reaction, formed from the reaction of MG with *N*-*α*-t-BOC-arginine (Gomes et al. 2005). AP residue has not been identified in plasma protein due to the lower concentration and/or heat labile of AP over 24 h 110°C treatment (Shipanova et al. 1997). The plasma concentrations of AP-free adduct did not show significant differences between SD and SDT rats at same ages or within each group over the duration of investigation, with much lower levels in rat plasma (Fig.4E).

An important effect of AGEs on protein structure and function is the formation of nonsulfhydryl cross-links. Pentosidine residues are a widely studied fluorescent AGE cross-link of plasma protein (Thornalley 2005b). The AGE competitive ELISA kit used an anti-AGE polyclonal antibody that detects multiple AGE structures mainly including CML and pentosidine, but not CEL or methylglyoxal-derived AGE residue in protein samples. As shown in Table1, the AGE levels in plasma protein of SD and SDT rats determined by AGE competitive ELISA assay did not show the significant differences over the duration of investigation.

## Discussion

The present study has revealed significant increase in MG-H1 residue content of plasma protein at the prediabetic stage of a spontaneously diabetic rat model for the first time, irrespective of unaltered fasting blood glucose and constant plasma levels of other AGEs.

Studies on AGEs have been mainly focused on their role in diabetes mellitus and complications, and the plasma levels of some AGEs (e.g., MG-H1 and CML) in diabetic patients have been reported to be much higher than those in normal subjects (Ahmed et al. 2005). However, an association of glycation metabolites with prediabetes, a high-risk state of developing diabetes, has not been investigated intensively due to the lack of appropriate animal models and high-sensitive detection assays of related metabolites. Our study got the first insight into the chronic variation in quantitatively important metabolites of protein glycation in a spontaneously diabetic rat model during the pathogenic progress of diabetes. This is also the first application of our proposed high-sensitive TNBS-MS method which aided by stable isotopic IS to quantify a comprehensive range of AGE adducts in plasma samples of long-term living animals.

AGE-free adducts in plasma are consistent with their release from damaged proteins by proteolysis (Thornalley 2005a) and clearance by renal dialysis (Agalou et al. 2005). Besides, other sources of AGE-free adducts, such as digestion of damaged protein in food, should be taken into account (Uribarri et al. 2010). In the present study, AGE-free adducts showed relatively stable behavior until the age of 24 weeks in both SD and SDT rats. The marked increase in MG-H1- and CML-free adducts were expected at the age of 24 weeks, given the marked increased fasting BGL at diabetic stage consideration. CEL- and AP-free adducts in plasma of SDT rats, however, were unexpectedly exhibited similar levels at the age of 24 weeks, with respect to the age-matched SD rats. Considering that CEL and AP shared the same precursor MG with MG-H1, the fourfold increase in the level of MG-H1-free adduct may contribute to the invariable levels of CEL- and AP-free adducts in SDT rats. An enhanced clearance of AGEs via hyperfiltration of renal excretion may serve as a further contribution to this unexpected result (Karachalias et al. 2010). Interestingly, G-H1-free adduct showed a comparable level with SD control rats over the duration of investigation, suggested that formation of AGE-free adducts in vivo may be affected by environmental conditions such as temperature and humidity, in the similar way as AGE formation in food (Goldberg et al. 2004). This study revealed that these five typical AGE-free adducts, which can be affected by several factors and released after a relative long duration, are unlikely served as predictors of prediabetes. Besides, not every AGE-free adduct will be increased at the onset of diabetes due to the different rates of metabolism.

Increased protein content of AGE residues in diabetes leads to the impairment of cellular and plasma protein function (Ahmed and Thornalley 2003). The G-H1, CEL, and CML residues of plasma protein of SD control rats in present study were consistent with previously reported contents in normal SD rats (Karachalias et al. 2010), and showed increased accumulation in plasma protein in SDT rats at diabetic stage (24-week-old). Some tiny and unmarked decrease tendency of AGE residues indicated that there is limited direct elimination of glycated proteins by clearance and excretion of corresponding free adducts after proteolysis (Ahmed et al. 2004). In SDT rats, MG-H1 residue contents of plasma protein were increased significantly since the prediabetic stage (16-week-old) and generally unchanged after this increase, meanwhile in SD rats the contents did not show any increased tendency over the duration of the investigation. MG-H1 residue of plasma protein, a major quantitatively AGE residue, were reported to be associated with increased cardiovascular disease mortality in nondiabetic women (Kilhovd et al. 2009), and retinopathy in patients with type 2 diabetes (Kilhovd et al. 2003). The present study strongly demonstrated that MG-H1 residue of plasma protein may also play a role in the developing of prediabetes in a spontaneously diabetic rat model and can be considered as a potent predictor of prediabetes.
